# Supplementary material for: Effectiveness and Safety of Ayurvedic Medicines in Type 2 Diabetes Mellitus Management: A Systematic Review and Meta-Analysis
Source: Front Pharmacol. 2022 Jun 8;13:821810. doi: 10.3389/fphar.2022.821810 (PMC9213670; doi:10.3389/fphar.2022.821810)
Supplement: Supplementary file 1 [file DataSheet1.zip › Supplementary Material/Forest and Funnel Plots/Punica granatum/TG.pdf]

Punica granatum - TG

Study	Intervention N	Intervention Mean	Intervention SD	Comparator N	Comparator Mean	Comparator SD	Mean Diff. with 95% CI	Weight (%)
Faghihimani 2017	37	157.00	91.00	37	132.00	62.00	25.00 [-10.48, 60.48]	28.69
Khajebishak 2019a, Khajebishak 2019b	26	160.00	62.20	26	162.90	37.60	-2.90 [-30.84, 25.04]	39.89
Grabež 2020	19	206.21	204.43	18	186.74	77.00	19.47 [-81.18, 120.12]	4.62
Hashemi 2020	30	151.24	67.94	30	172.78	78.45	-21.54 [-58.68, 15.60]	26.80
**Overall**							1.14 [-20.97, 23.25]	

Heterogeneity: $\tau^2 = 115.83$, $I^2 = 22.26\%$, $H^2 = 1.29$

Test of $\theta_i = \theta_j$: $Q(3) = 3.38$, $p = 0.34$

Test of $\theta = 0$: $z = 0.10$, $p = 0.92$

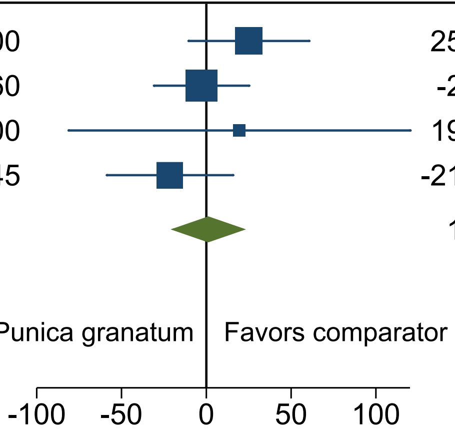

Random-effects REML model
